# Evaluation of homogenization methods for seasonal snow depth data in the Austrian Alps, 1930–2010

**DOI:** 10.1002/joc.6095

**Published:** 2019-04-30

**Authors:** Giorgia Marcolini, Roland Koch, Barbara Chimani, Wolfgang Schöner, Alberto Bellin, Markus Disse, Gabriele Chiogna

**Affiliations:** ^1^ Department of Civil Environmental and Mechanical Engineering University of Trento Trento Italy; ^2^ Faculty of Civil, Geo and Environmental Engineering Technical University of Munich Munich Germany; ^3^ Department of Climate Research, Central Institute for Meteorology and Geodynamics (ZAMG) Vienna Austria; ^4^ Department of Geography and Regional Science University of Graz Graz Austria; ^5^ Faculty of Geo‐ and Atmospheric Sciences University of Innsbruck Innsbruck Austria

**Keywords:** Alps, Austria, homogenization, HOMOP, INTERP, PRODIGE, SNHT, snow

## Abstract

Despite the importance of snow in alpine regions, little attention has been given to the homogenization of snow depth time series. Snow depth time series are generally characterized by high spatial heterogeneity and low correlation among the time series, and the homogenization thereof is therefore challenging. In this work, we present a comparison between two homogenization methods for mean seasonal snow depth time series available for Austria: the standard normal homogeneity test (SNHT) and HOMOP. The results of the two methods are generally in good agreement for high elevation sites. For low elevation sites, HOMOP often identifies suspicious breakpoints (that cannot be confirmed by metadata and only occur in relation to seasons with particularly low mean snow depth), while the SNHT classifies the time series as homogeneous. We therefore suggest applying both methods to verify the reliability of the detected breakpoints. The number of computed anomalies is more sensitive to inhomogeneities than trend analysis performed with the Mann–Kendall test. Nevertheless, the homogenized dataset shows an increased number of stations with negative snow depth trends and characterized by consecutive negative anomalies starting from the late 1980s and early 1990s, which was in agreement with the observations available for several stations in the Alps. In summary, homogenization of snow depth data is possible, relevant and should be carried out prior to performing climatological analysis.

## 1 INTRODUCTION

Historical time series of meteorological variables represent a precious and fundamental source of information for environmental studies today. They are used as input data for models, as validation datasets as well as for the identification of trends and changes in the dynamic of natural systems. It is therefore important to apply and develop appropriate tools to evaluate the quality of the available time series. Among the various tests performed to assess the quality of historical time series, the homogeneity test has received significant attention in climate change studies. Homogenization techniques aim to detect, and when possible adjust, the changes in a time series that cannot be attributed to natural variations or to climate change (Aguilar *et al.,* 2003). The change of the measurement equipment or the relocation of the measuring station are typical examples of factors that can affect the interpretation of collected data and make a time series inhomogeneous. Homogenization of time series consists of two steps: breakpoint detection and the following adjustment thereof. Several techniques have been developed for the homogenization of time series such as the standard normal homogeneity test (SNHT) (Alexandersson, 1986; Alexandersson and Moberg, 1997), HOMOP (Nemec et al. 2013) (which applies PRODIGE [Caussinus and Mestre, 2004] for breakpoint detection and INTERP (Vincent, 2002) for the calculation of adjustments), the two‐phase regression method (Easterling and Peterson, 1995), HOMogenizaton softwarE in R (Mestre *et al.,* 2013), and the multiple linear regression method (Vincent, 1998). Peterson *et al*. (1998) and Aguilar *et al*. (2003) provide a comprehensive review of these homogenization algorithms. Vincent (1998), Ducré‐Robitaille *et al*. (2003), Reeves *et al*. (2007), Venema *et al*. (2012) and others demonstrated the importance of comparing the homogenization results obtained by applying different homogenization algorithms in order to highlight their strengths and weaknesses.

Most of these homogenization algorithms have been developed for and applied to temperature and precipitation time series. The homogenization of snow depth time series had rarely been attempted. The SNHT was used to test the homogeneity of snow depth time series in the Province of Trento (Marcolini *et al.,* 2017a) and in the Adige catchment (Marcolini *et al.,* 2017b). The PRODIGE method was used to analyse time series in Austria (Koch *et al.,* 2014; Schöner *et al.,* 2019). Further applications of homogenization methods to snow depth time series were also reported by Brown and Braaten (1998). Due to the small number of studies, it is therefore important to deepen our knowledge on the homogenization of snow depth time series. Obtaining high‐quality historical snow depth time series is of particular interest for a variety of applications such as studies dealing with water resources (Beniston *et al.,* 2003; Barnett *et al.,* 2005; Beniston, 2006), hydrology (Tuo *et al.,* 2018a; 2018b), winter tourism (Koenig and Abegg, 1997) and hydropower production (Beniston, 2012). Moreover, snow depth, which has been measured by the national hydrological and meteorological networks since the 19th century, is an important indicator for climatological studies in alpine areas (Beniston *et al.,* 1997; Barnett *et al.,* 2005; Marty, 2008; Scherrer *et al.,* 2013). Snow depth is strongly influenced by temperature and precipitation changes (Beniston *et al.,* 2003; Bartlett *et al.,* 2004; Barnett *et al.,* 2005; Beniston, 2006; Kim *et al.,* 2013) at large, synoptic spatial scales and additionally by wind at local scales. Moreover, snow depth is sensitive to other morphological factors such as hillslope orientation and elevation. For this reason, snow depth is also an indicator of climate change in relationship to the morphology of the region (Beniston *et al.,* 2018). The complexity of snow dynamics makes the homogenization of snow depth time series a challenging task (Begert *et al.,* 2008).

In the present study, we compare the performances of the SNHT and HOMOP homogenization algorithms by applying them to an Austrian snow depth time series dataset. These two methods were selected as they were recently used for the same purpose in the literature (Marcolini *et al.,* 2017a; Schöner *et al.,* 2019). To filter out the high‐frequency fluctuations of snow depth data on a short (i.e., daily) time scale, the breakpoint detection and the computation of the adjustment factors are based on seasonal data (i.e., mean snow depth value computed between November and March). We also investigate if the detected inhomogeneities are significant for the interpretation of anomalous behaviours observed in the dataset.

## 2 DESCRIPTION OF THE DATASET

Snow depth observations in Austria on a daily time scale were collected by the Zentralanstalt für Meteorologie und Geodynamik (ZAMG) and the Hydrographisches Zentral Bureau (HZB). Individual station datasets cover a time period spanning from the late nineteenth century until the present day. The station density varies from region to region due to the presence of complex mountainous terrain.

The spatial representativeness of ground‐based measurements is limited, especially in regions of complex terrain where precipitation and temperature are highly variable both in time and space. In addition, large uncertainty in snow observations is often found due to the transport of snow by wind, particularly in mountainous regions (Sevruk, 1986; Rasmussen *et al.,* 2012).

**Figure 1:**
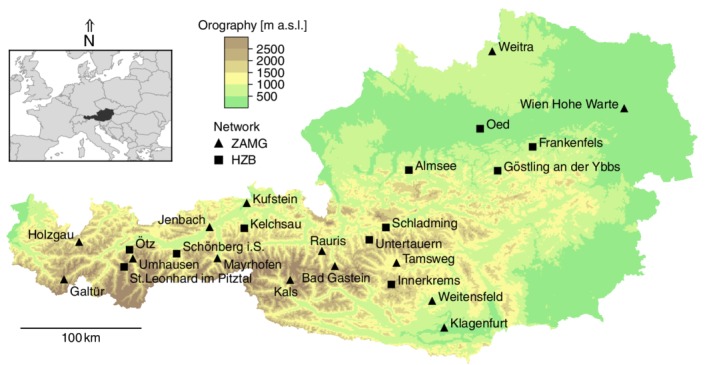
Map of the stations used for the intercomparison experiment. Reference stations for the homogenization are not shown. The black triangles (squares) represent stations in the ZAMG (HZB) observational network

In the present study, we test the homogeneity of a set of snow depth time series collected at 25 meteorological stations (14 ZAMG and 11 HZB stations), shown in Figure 1 and detailed in Table 1. These stations are located in different representative climatic regions of Austria, and the mean length of the snow depth time series is roughly 73 years. The elevations of the selected stations range from 198 m (station Wien Hohe Warte) to 1,577 m a.s.l. (station Galtür). Each time series consists of manual snow depth measurements taken at 07 : 00 a.m. Central European Time, ensuring that the dataset is not affected by changes in the measurement methods as seen in other alpine regions (e.g., Marcolini *et al.,* 2017a).

**Table 1:** Name, temporal length, location and data provider of the stations to be checked for homogeneity

Name	Temporal length	Temporal length	Location	Location	Data provider
Galtür	January 1, 1936	December 31, 2011	47.57527	12.16277	ZAMG
Innerkrems	January 1, 1896	July 31, 2011	46.97194	13.75056	HZB
Kals	May 1, 1951	December 31, 2011	47.00472	12.64638	ZAMG
St. Leonhard im Pitztal	January 1, 1930	December 30, 2011	47.07611	10.83889	HZB
Holzgau	January 1, 1936	December 31, 2011	47.25000	10.33333	ZAMG
Bad Gastein	January 1, 1948	December 31, 2009	47.11056	13.13333	ZAMG
Umhausen	January 1, 1936	December 31, 2011	47.14250	10.92889	ZAMG
Tamsweg	January 1, 1949	June 30, 2008	47.13306	13.80833	ZAMG
Schönberg im Stubaital	January 1, 1926	December 30, 2011	47.18389	11.40083	HZB
Untertauern	April 17, 1896	December 28, 2011	47.30556	13.50889	HZB
Rauris	January 1, 1895	March 31, 2011	47.22361	12.99250	ZAMG
Kelchsau	July 1, 1895	December 30, 2011	47.38639	12.13889	HZB
Ötz	May 1, 1910	August 31, 2012	47.20583	10.88611	HZB
Schladming	January 1, 1961	December 30, 2011	47.39833	13.69528	HZB
Weitensfeld	January 1, 1953	December 31, 2011	46.84917	14.19083	ZAMG
Mayrhofen	January 13, 1936	December 31, 2011	47.15944	11.85056	ZAMG
Almsee	January 1, 1961	December 30, 2011	47.82398	13.95083	HZB
Weitra	December 1, 1930	December 31, 2011	48.70222	14.89861	ZAMG
Jenbach	April 1, 1955	December 31, 2008	47.38889	11.75806	ZAMG
Göstling an der Ybbs	September 1, 1956	November 30, 2012	47.81056	14.93139	HZB
Kufstein	January 1, 1936	December 31, 2011	47.57528	12.16278	ZAMG
Frankenfels	September 1, 1960	November 30, 2012	47.98222	15.32389	HZB
Klagenfurt	April 1, 1950	December 31, 2011	46.64833	14.31833	ZAMG
Oed	August 31, 1958	December 30, 2011	48.12278	14.74417	HZB
Wien Hohe Warte	January 1 1916	July 31, 2012	48.24861	16.35639	ZAMG

An extensive quality control was performed on the ZAMG network time series, including tests for internal and spatial consistency, respectively. Internal consistency was evaluated by checking observations of different parameters at the same station such as the temperature, new snow sum and snow measurement, which should be consistent with precipitation. Spatial consistency was tested by checking the snow observations from different stations in the near vicinity. Quality control for most HZB measurements began in the 1970s, and the raw digitized HZB data were subject to plausibility checks (e.g., selected internal and external consistency checks depending on data availability) for the period before 1970 to reject major errors in the time series.

A detailed metadata history exists for the ZAMG stations and includes descriptions of station relocations and changes in the observation systems, for example, changes of the observer. In contrast, only metadata on the stations' relocations are available for the HZB stations. Since systematic errors in observations are some of the main error sources in subsequent trend analyses, it is implied that the time series of stations that underwent major relocations in the past may poorly represent “true” temporal variability. With regards to the reliability of the snow data, the observational error is assumed to be small compared to artificial shifts in snow depth time series caused by the station relocations (e.g., Rasmussen *et al.,* 2012; Terzago *et al.,* 2013).

**Figure 2:**
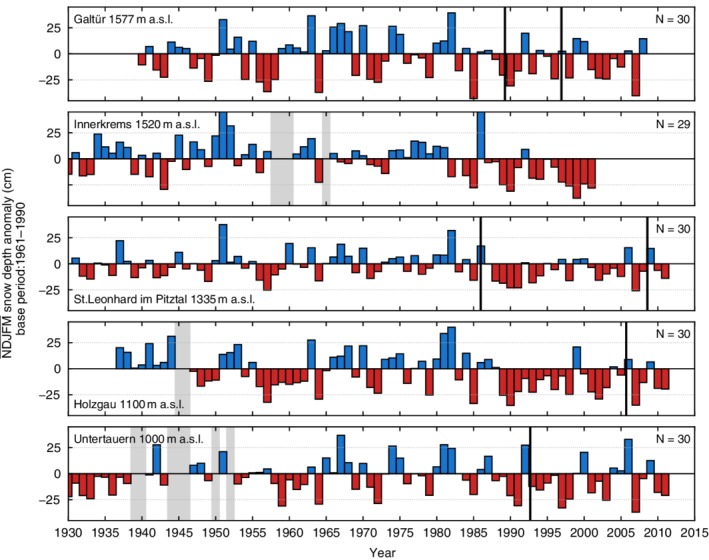
Mean seasonal snow depth anomalies (cm) at five stations (ZAMG, HZB) above 1,000‐m elevation with respect to the 1961–1990 base period. The time series are ordered by elevation, with the highest on top. Anomalies are calculated using the unhomogenized data. Black vertical lines indicate a documented station relocation, grey shaded areas represent the location of missing data in the time series. The number of valid seasonal observations *N* (years) in the range of the base period used is shown in the top right corner

**Figure 3:**
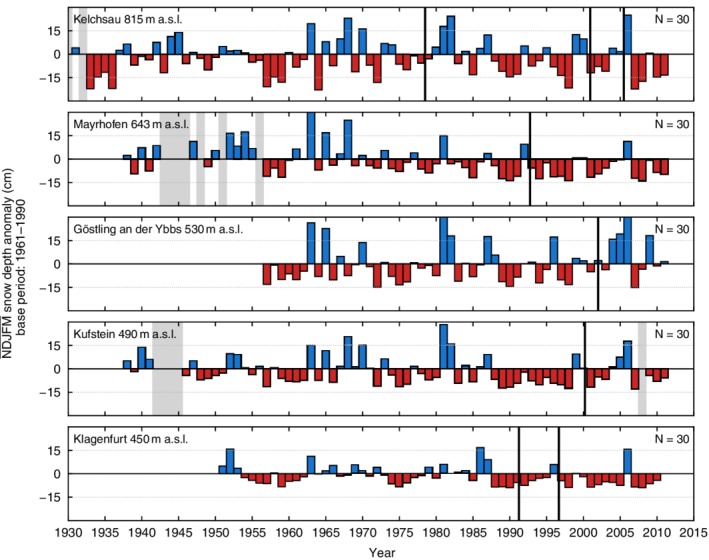
Same as Figure 2, but for five stations below 1,000‐m elevation. The range of the snow depth anomalies is adjusted

Figures 2 and 3 show the mean seasonal snow depth anomalies of five high (above 1,000 m a.s.l.) and five low (below 1,000 m a.s.l.) elevation stations within the dataset with respect to a 1961–1990 base period. It can be seen that the anomalies display both large spatial and temporal variability. Closer inspection reveals a general increase in consecutive negative anomalies starting from the late 1980s and early 1990s, which was also observed by Marty (2008) and Reid *et al*. (2016). The computation was performed using the initial time series prior to performing the homogenization of the dataset. Most of the stations have undergone relocation, introducing possible biases that may yield inhomogeneities in the time series and therefore influence the anomalies shown in Figures 2 and 3.

## 3 DESCRIPTION OF THE METHODS

### 3.1 PRODIGE

The PRODIGE method for breakpoint detection was developed by Caussinus and Mestre (2004), and it is integrated in the software package HOMOP (Nemec et al.; 2013). In contrast to other homogenization algorithms (Alexandersson, 1986; Easterling and Peterson, 1995), the PRODIGE method does not require the creation of a synthetic reference time series representative of the climatic area of the tested (candidate) time series (cf. the following paragraph describing the creation of a reference time series when applying the SNHT method). Instead, the PRODIGE algorithm defines the interval in each time series between two change points as reliable homogenous segments. All of these homogeneous sections are used as reference series. The tested time series is then compared to all other series within the same climatic area by calculating a series of ratios between the tested and each reference series. This is analogous to the application of the method commonly applied for the homogenization of precipitation time series. These ratio series are then tested for discontinuities, and the detection of the discontinuities is based on a penalized log‐likelihood criterion (Mestre *et al.,* 2013) to overcome the increased likelihood of detection with an increased number of homogeneous segments (overfitting). The penalized log‐likelihood L can be written as:

(1) $$L=L_{K}-\beta^{*}\mathrm{pen}\left(K\right),$$

where *L*_*K*_ is the maximum log‐likelihood of the best partitioned time series into *K* segments, *β* is a coefficient of penalization and pen(*K*) is a penalty criterion that increases with increasing number of segments (Picard *et al.,* 2005). Thus, the final estimated number of breakpoints maximizes Equation 1. In order to find the optimum number of breakpoints, penalizing criteria suggested by Caussinus and Lyazrhi (1997), Jong *et al*. (2003) and Lebarbier (2005) are used in the detection process.

The first step in applying PRODIGE consists of identifying the set of time series that belong to the same climatic area of the tested time series. For this purpose, the following criteria are applied: (a) the correlation coefficient between tested and reference time series is larger than 0.7 when considering daily values; (b) Only reference stations within a horizontal radius of 100 km centred on the tested station are considered; and (c) a maximum vertical difference between the stations where the time series are recorded is less than 300 m. In the present study, the number of reference stations considered for each tested time series is on average equal to 12, though not all covered the whole observational period of the tested station. The second step consists of the assignment of the breakpoint detected in the ratios time series to one of the two considered time series. In fact, it is unknown a priori which one of the two time series causes the breakpoint identified in the ratios time series. If a detected change point remains constant throughout the set of comparisons of a tested time series with its neighbours, the breakpoint can be attributed to the tested station (Caussinus and Mestre, 2004).

The procedure is applied to the mean seasonal snow depth time series computed using the daily snow depth data records. Two mean snow depth time series are considered: the first is the average computed considering the periods from December to February, while the second considers the period from November to March (NDJFM). Thus, the ratios between the mean seasonal snow depth series of the tested time series and the highly correlated reference time series are calculated. For each of the three penalizing criteria considered in the maximum log‐likelihood estimate, the breakpoint is considered reliable if it appears in more than half of the reference time series. Moreover, breaks were only considered, if they were detected by at least two of the penalizing criteria in both seasons. If necessary, the location of the breakpoint in time was adjusted according to the available station meta‐information.

Once breakpoints were detected, a modified version of the INTERP method (Vincent et al. 2002) was applied to adjust inhomogeneities in the daily snow depth data records. Since ground‐based snow depth measurements are characterized by high spatial and temporal variability due to a number of influencing factors including temperature, wind, radiation, which could not be accounted for using the adjustment factor, a simple approach was chosen, for which a reasonable improvement of the time series can be expected. The computation of a constant adjustment factor is therefore based on a seasonal (NDJFM) scale. The equation for calculating the seasonal adjustment for the best correlated reference station has the form:

(2) $$\text{adjustment factor}=\frac{\text{median}\left\{\frac{C_{2}}{R_{2}}\right\}}{\text{median}\left\{\frac{C_{1}}{R_{1}}\right\}},$$

where indices 1 and 2 represent the time period after and before the detected inhomogeneity and *C* (*R*) is the accumulated seasonal (NDJFM) snow depth of the candidate (reference) time series. The final step involves the multiplication of the inhomogeneous daily scale sub–period before the breakpoint by the seasonal adjustment factor.

### 3.2 The standard normal homogeneity test

The SNHT was introduced by Alexandersson (1986). The first step of the SNHT is the creation of a standardized ratio series **Z** between the tested time series **Y** and a reference time series **X** that should be representative of the climatic area of **Y**.

The hypothesis *H*_0_ that the time series **Y** is homogeneous and its alternative hypothesis *H*_1_ that **Y** contains a breakpoint at time step *a* can be formulated as shown in 3, 4, respectively:

(3) $$H_{0}:\;Z_{i}\in N\left(0,1\right)\;i\in \left\{1,\ldots ,n\right\},$$

(4) $$H_{1}:\;\left\{\begin{array}{l} Z_{i}\in N\left(\mu_{1},1\right)\;i\in \left\{1,\ldots ,a\right\}\\ Z_{i}\in N\left(\mu_{2},1\right)\;i\in \left\{a+1,\ldots ,n\right\} \end{array}\right.,$$

where *N*(*μ*, *σ*^2^) denotes the normal distribution with mean *μ* and *SD σ*, *n* is the length of the time series **Z** and **Z**_*i*_ indicates the value of **Z** at time step *i*.

We then study the test statistic *T*_max_ defined as

(5) $$T_{\max}=\max_{1\leq a\leq n-1}\left\{T\left(a\right)\right\}=\max_{1\leq a\leq n-1}\left\{a{\bar{z_{1}}}^{2}+\left(n-a\right){\bar{z_{2}}}^{2}\right\},$$

where $\bar{z_{1}}$ and $\bar{z_{2}}$ are the arithmetic averages of the time series **Z** up to the time step *a* and from the time step *a* + 1 to the end, respectively. If *T*_max_ is above a critical level (Khaliq and Ouarda, 2007), depending on the length of the time series **Z** and on the chosen confidence level of a detected breakpoint (which in our case is set to *α* = 0.95), the data point *a* corresponding to *T*_max_ is taken as the breakpoint.

The reference time series **X** is constructed starting from *k* reference stations **S**_*j*_, where *j* = 1 … *k*. The reference stations fulfil specific requirements as stated in Marcolini *et al*. (2017a). This implies that the homogenization analysis is performed on the period in which **X** can be defined. Marcolini *et al*. (2017a) suggest constructing the reference time series following two different approaches (Peterson and Easterling, 1994; Alexandersson and Moberg, 1997) in the case of mean seasonal snow depth time series. A breakpoint is considered reliable if the detection is agreed upon between the two time series or when there is high statistical significance. The main difference between these two approaches is the following. According to Alexandersson and Moberg (1997), we directly create the reference time series starting from the raw values of the reference stations. When using the approach proposed by Peterson and Easterling (1994), we instead construct the reference time series starting from the time series of the temporal increments of the reference stations.

In Marcolini *et al*. (2017a), the choice of the reference stations **S**_*j*_ was performed on all available time series of the investigated region. In the present work, instead, the reference stations are chosen among the set of stations used to perform the homogeneity test using the PRODIGE method. This allows for a more consistent comparison between the two methods.

A detected breakpoint *a* is adjusted by multiplying the tested time series **Y** by an adjustment factor cf from the first recorded time stamp until the breakpoint *a*. The adjustment factor *cf* for the mean seasonal snow depth time series is computed as

(6) $$\mathrm{cf}=\frac{{\bar{q}}_{2}}{{\bar{q}}_{1}},$$

with ${\bar{q}}_{1}$ and ${\bar{q}}_{2}$ defined as follows:

(7) $${\bar{q}}_{1}=\sigma_{Q}{\bar{z}}_{1}+\bar{Q}$$

(8) $${\bar{q}}_{2}=\sigma_{Q}{\bar{z}}_{2}+\bar{Q},$$

where *σ*_**Q**_ and $\bar{Q}$ are the *SD* and the mean of the non‐standardized ratio time series **Q** = **X**/**Y** (Alexandersson and Moberg, 1997).

## 4 RESULTS AND DISCUSSION

### 4.1 Breakpoint detection

As shown in Table 2, in 56% of the cases, the two algorithms agreed in their analyses of the time series, which meant both classified the series as homogeneous, or identified the same breakpoints. The two algorithms were said to have identified the same breakpoint if the difference between the time location of the detected breakpoints was 2 years or less. In fact, SNHT as well as other homogenization methods has some level of uncertainty in the identification of the correct temporal location of the breakpoint, as also discussed in Marcolini *et al*. (2017a) and Lindau and Venema (2016). Moreover, the temporal location of breakpoints identified by PRODIGE was sometimes adjusted using metadata. In eight cases (Innerkrems, Kals, Holzgau, Umhausen, Mayrhofen, Wien Hohe Warte, Weitensfeld and Weitra), PRODIGE detected a suspicious breakpoint. The breakpoints identified in six time series (Innerkrems, Kals, Holzgau, Umhausen, Mayrhofen and Wien Hohe Warte) were considered suspicious due to low snow depth. In fact, they all occurred in winter seasons with low mean snow depths (beginning of the 1970s, end of the 1980s, beginning of the 1990s, see also Marty, [2008]) and they are not considered reliable breaks since they cannot be linked to changes in the observational environment. The detection algorithm PRODIGE is more sensitive than the SNHT to changes at low snow depths. The breakpoints found by PRODIGE in the time series of Weitensfeld and Weitra were classified as suspicious because they were close to the end of the tested time series (1 and 3 years from the end, respectively). Hence, these eight break points were not adjusted by PRODIGE. In the six cases where the suspicious breakpoints were caused by low mean seasonal snow depth, the time series were classified as homogenous by the SNHT. Since the results would not require the application of an adjustment factor, we consider these results in the cases for when the two methods agree. In conclusion, the two methods reached consistent results since both suggest the same action (i.e., to adjust or not to adjust the time series) in 80% of the stations.

**Table 2:** Results of the homogeneity analysis

Site	Elevation (m a.s.l.)	Results		Adjustment factors	
		PRODIGE	SNHT	INTERP	SNHT
Galtür	1,577	1988	1987	1.22	1.24
Innerkrems	1,520	Y	–		
Kals	1,352	Y	–		
St. Leonhard im Pitztal	1,335	1985	–	0.95	
Holzgau	1,100	Y	–		
Bad Gastein	1,092	1972	1974	1.38	1.84
Umhausen	1,041	Y	–		
Tamsweg	1,026	1983	–	0.83	
		1998	–	0.73	
Schönberg im Stubaital	1,005	–	–		
Untertauern	1,000	–	–		
Rauris	934	1973	1973	1.57	2.18
		1993	1995	1.64	1.58
Kelchsau	815	–	–		
Ötz	760	–	–		
Schladming	730	–	–		
Weitensfeld	704	Y	1998		0.66
Mayrhofen	643	Y	–		
Almsee	590	–	–		
Weitra	572	Y	1975		0.50
Jenbach	530	–	–		
Göstling an der Ybbs	530	–	2001		1.36
Kufstein	490	–	–		
Frankenfels	465	–	–		
Klagenfurt	450	–	–		
Oed	400	1996	1996	1.22	1.81
Wien Hohe Warte	198	Y	–		

*Note*: In the cases in which the result is indicated with Y, PRODIGE‐detected suspicious breaks.

The analysis of the sites St. Leonhard im Pitztal, Tamsweg, Weitra, Weitensfeld and Göstling an der Ybbs shows different results for the two methods. We did not find any correlation between the altitude of the site or its position and the difference in the results of the algorithms. Moreover, as discussed later, we were able to consider the difference between the SNHT result (homogeneous series) and the PRODIGE result of little practical importance when considering that the adjustment factor computed for St. Leonhard im Pitztal by PRODIGE is equal to 0.95.

**Figure 4:**
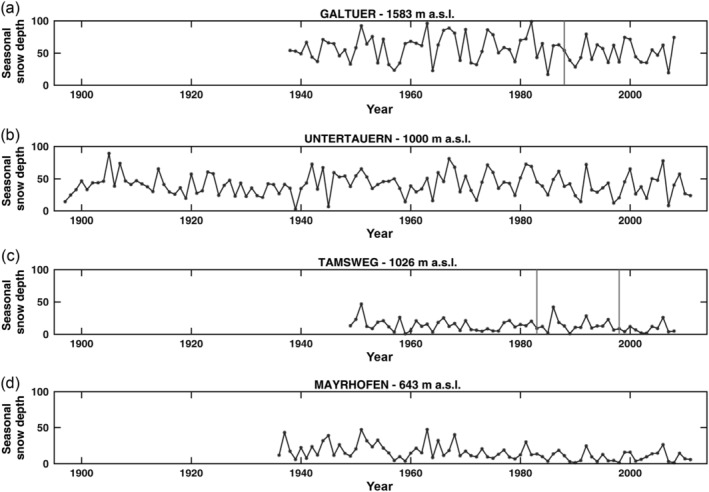
Example of the time series used for the intercomparison experiment. In the upper panel (a), a time series where both the used algorithm found the same unique breakpoint is shown. The time series of Untertauern (b) is homogeneous according to the results of both the algorithms. The third plot (c) shows a time series where PRODIGE and SNHT found different breakpoints. Finally, in plot (d) is shown an example of a time series which was classified as homogenous by the SNHT and as suspicious by PRODIGE (suspecious breakpoint in 1993)

In Figure 4, four time series are shown. The first plot shows the time series of Galtür, where both homogenization methods detect the same breakpoint. The second plot shows the time series of Untertauern, where both algorithms classified the time series as homogeneous. The time series for Tamsweg (Figure 4c) saw different results from the two homogenization methods. Finally shown in Figure 4d are the time series of Mayrhofen, which are classified as homogeneous by the SNHT and as suspicious by PRODIGE.

### 4.2 Adjustment of the inhomogeneities

As described in Section 3, the adjustment of a breakpoint was performed by multiplying the first part of the time series from the beginning up to the breakpoint by an adjustment factor. Due to the multiplicative approach, it is only possible to modify the registered snow depth but not to add new snow days to the adjusted time series. The adjustment factors of the time series where the two algorithms detected breakpoints (Bad Gastein, Galtür, Rauris and Oed) are in agreement (see Table 2), that is, in both cases they indicate an adjustment factor larger than 1. However, we observe that on average the deviation between the two adjustment factors is 20%, with SNHT values being generally larger than the ones computed using INTERP. Note that the SNHT is primarily intended to be applied for the detection and adjustment of one single breakpoint. It is well known (Alexandersson and Moberg, 1997) that its performance in the detection and adjustment of multiple breakpoints as seen in the station Rauris is generally poor, particularly for early breaks.

There are several possible reasons explaining the variability in the computed adjustment factors. First, the two mathematical expressions reported in Equation 2 and Equation 6 are not equivalent. The second and probably more important factor is that the candidate station is not necessarily compared to the same set of reference stations, since the procedures used by the two methods to select them are different (see 3.1, 3.2).

### 4.3 Identification of the cause for the breakpoint

Metadata can be used to find the cause of a breakpoint in a time series. As described above, our dataset consists entirely of data that have been collected manually using the same procedure throughout the entire period. Therefore, the most important metadata are those concerning the relocation of the station given in Table 3. As can be deduced from the table, most of the stations were relocated several times over their history. Still, the number of detected breakpoints was relatively low in comparison to the number of relocations that occurred in the past. This means that relocation of a station does not always cause a statistically significant breakpoint in the time series. However, all breakpoints detected using the PRODIGE method that were not classified as suspicious correspond to station relocations. For results obtained using the SNHT method, only the breakpoints detected for Weitra and Weitensfeld are not associated with station relocations. This result is not indicative of a poorly performing statistical test since the metadata may be incomplete and does not include other sources of possible breakpoints such as a change in the operator taking the measurement.

**Table 3:** Metadata of station relocation for the dataset

Site	Latitude	Longitude	Elevation (m)	Start date	End date	Environment
*Galtür*	46.96667	10.18333	1,583	‐	March 31, 1989	Narrow valley, village
	46.96444	10.19361	1,648	**April 1, 1989**	November 30, 1996	Narrow valley, village boundary
	46.96806	10.18556	1,577	December 1, 1996	August 31, 2008	Narrow valley, village
	46.96806	10.18556	1,587	September 1, 2008	December 31, 2011	‐
Kals	47.00000	12.65000	1,347	‐	June 30, 1984	Valley, village
	47.00000	12.63333	1,350	July 1, 1984	May 31, 1992	‐
	47.00333	12.64611	1,338	May 1, 1993	December 31, 2007	‐
	47.00472	12.64639	1,352	January 1, 2008	December 31, 2011	‐
*St. Leonhard im Pitztal*	47.06778	10.86611	1,370	‐	December 16, 1985	Valley, village boundary
	47.07667	10.83667	1,335	**December 17, 1985**	July 28, 2008	Valley, village
	47.07611	10.83889	1,329	July 29, 2008	December 30, 2011	‐
Holzgau	47.26250	10.34917	1,100	‐	September 16, 2005	Valley, village
	47.26250	10.34222	1,080	September 17, 2005	December 31, 2011	‐
*Bad Gastein*	47.11667	13.13333	1,082	‐	December 31, 1975	Valley, village
	47.11667	13.13333	1,100	January 1, **1976**	October 31, 1995	‐
	47.09278	13.12083	1,100	November 1, 1995	December 31, 2009	‐
	47.11056	13.13333	1,092	January 1, 2010	March 31, 2011	‐
Umhausen	47.13750	10.93389	1,041	‐	September 30, 2003	Valley, village
	47.13917	10.92889	1,041	October 1, 2003	December 31, 2011	‐
*Tamsweg*	47.11667	13.80000	1,019	‐	December 5, 1956	Valley, village boundary
	47.13333	13.83333	1,019	December 6, 1956	November 30, 1960	‐
	47.11667	13.80000	1,012	December 1, 1960	February 28, 1983	‐
	47.13333	13.80000	1,012	**March 1, 1983**	May 31, 1998	‐
	47.12472	13.81000	1,025	**June 1, 1998**	June 30, 2008	Valley, village
	47.13306	13.80833	1,026	July 1, 2008	March 31, 2011	‐
Schönberg im Stubaital	47.18333	11.40000	1,010	‐	February 15, 1993	Valley, village
	47.18750	11.40472	1,005	February 16, 1993	January 22, 2009	‐
	47.18389	11.40083	1,009	January 22, 2009	December 30, 2011	‐
Untertauern	47.30556	13.50861	1,000	‐	September 6, 1992	Valley, village
	47.30556	13.50889	1,000	September 7, 1992	December 28, 2011	‐
*Rauris*	47.21667	13.00000	945	‐	October 31, 1973	Valley, village boundary
	47.25000	13.00000	945	**November 1, 1973**	June 30, 1989	‐
	47.25000	12.98333	916	July 1, 1989	December 31, 1993	‐
	47.22361	12.99250	934	**January 1, 1994**	March 31, 2011	Valley, village
Kelchsau	47.38333	12.13333	760	‐	June 30, 1978	Valley, village
	47.38806	12.13333	815	July 1, 1978	11/06/2000	‐
	47.39528	12.13778	800	June 12, 2000	June 29, 2005	‐
	47.38639	12.13889	815	June 30, 2005	December 30, 2011	‐
Ötz	47.20361	10.90000	775	‐	July 21, 1980	Narrow valley, village
	47.20500	10.89139	765	July 22, 1980	June 17, 1998	‐
	47.20583	10.88694	760	June 18, 1998	May 4, 2010	‐
	47.20583	10.88611	760	May 5, 2010	August 31, 2012	‐
Schladming	47.39247	13.68639	740	‐	December 31, 2003	Valley, village
	47.39833	13.69528	730	January 1, 2004	December 30, 2011	‐
*Weitensfeld*	46.85000	14.20000	715	‐	August 31, 1985	Valley, village
	46.84444	14.19639	705	September 1, 1985	August 31, 2007	‐
	46.84917	14.19083	704	September 1, 2007	December 31, 2011	‐
Mayrhofen	47.15000	11.85000	643	‐	September 30, 1992	Valley, village
	47.15944	11.85056	643	October 1, 1992	December 31, 2011	‐
Almsee	47.76750	13.95556	590	‐	02/07/2007	Valley, village
	47.82398	13.95083	574	July 3, 2007	December 30, 2011	‐
*Weitra*	48.69806	14.89889	580	‐	July 31, 2003	Lowland, village boundary
	48.70222	14.89861	572	August 1, 2003	July 31, 2012	‐
Jenbach	47.38333	11.75000	530	‐	June 30, 1994	Valley, village
	47.38889	11.75806	530	July 1, 1994	December 31, 2008	‐
*Göstling an der Ybbs*	47.81944	14.93083	544	‐	January 1, 2002	Lowland, village boundary
	47.81056	14.93139	530	**January 3, 2002**	November 30, 2012	Lowland, village
Kufstein	47.57417	12.16389	492	‐	March 15, 2000	Valley, village
	47.57528	12.16278	490	March 16, 2000	December 31, 2011	‐
Klagenfurt	46.65000	14.33333	447	‐	March 31, 1991	Valley, city boundary, rural area
	46.65000	14.33333	447	April 1, 1991	August 31, 1996	‐
	46.64833	14.31833	450	September 1, 1996	December 31, 2011	‐
*Oed*	48.11472	14.74583	360	‐	December 31, 1995	Lowland, village boundary
	48.12278	14.74417	393	**January 1, 1996**	December 30, 2011	Lowland, village
Wien Hohe Warte	48.24861	16.35639	198	‐	December 31, 1992	City, high density residential area
	48.24861	16.35639	198	January 1, 1993	July 31, 2012	‐

*Note*: In italics, we indicate the stations for which at least a reliable breakpoint was detected. Bold values are used when the detected break is in agreement with the relocation, within an interval of maximum 5 years.

The information contained in Table 3 does not provide a detailed explanation of why the relocation of a station causes a breakpoint. For example, elevation is one of the most sensitive factors that affects mean seasonal snow depth; however, we observe that breakpoints occur even with no change in elevation (e.g., Tamsweg station), and that even in cases with large changes in elevation, no breakpoints are identified (e.g., 55 m for Kelchsau station). The breakpoints associated with station relocation often correspond to significant changes in the surrounding environment (e.g., station relocation from the village boundary with isolated low buildings or trees to areas moderately covered by low buildings, and change in the density of the residential area). The small size of the dataset means a causal relation between station relocation and occurrence of the breakpoint cannot be found. A physically based validation of the results provided by homogenization methods for mean seasonal snow depth time series should be attempted in future studies.

### 4.4 Implication for time series analysis

The adjustment of an inhomogeneous time series has an effect on climatological analysis. We compare, for example, the anomalies of three time series with those of the corresponding adjusted time series. Note that the application of an adjustment factor influences the mean of the time series, which is later used for the computation of the anomalies. As a consequence, the anomalies of the adjusted time series show changes not only before, but also after the location of the corrected breakpoint.

**Figure 5:**
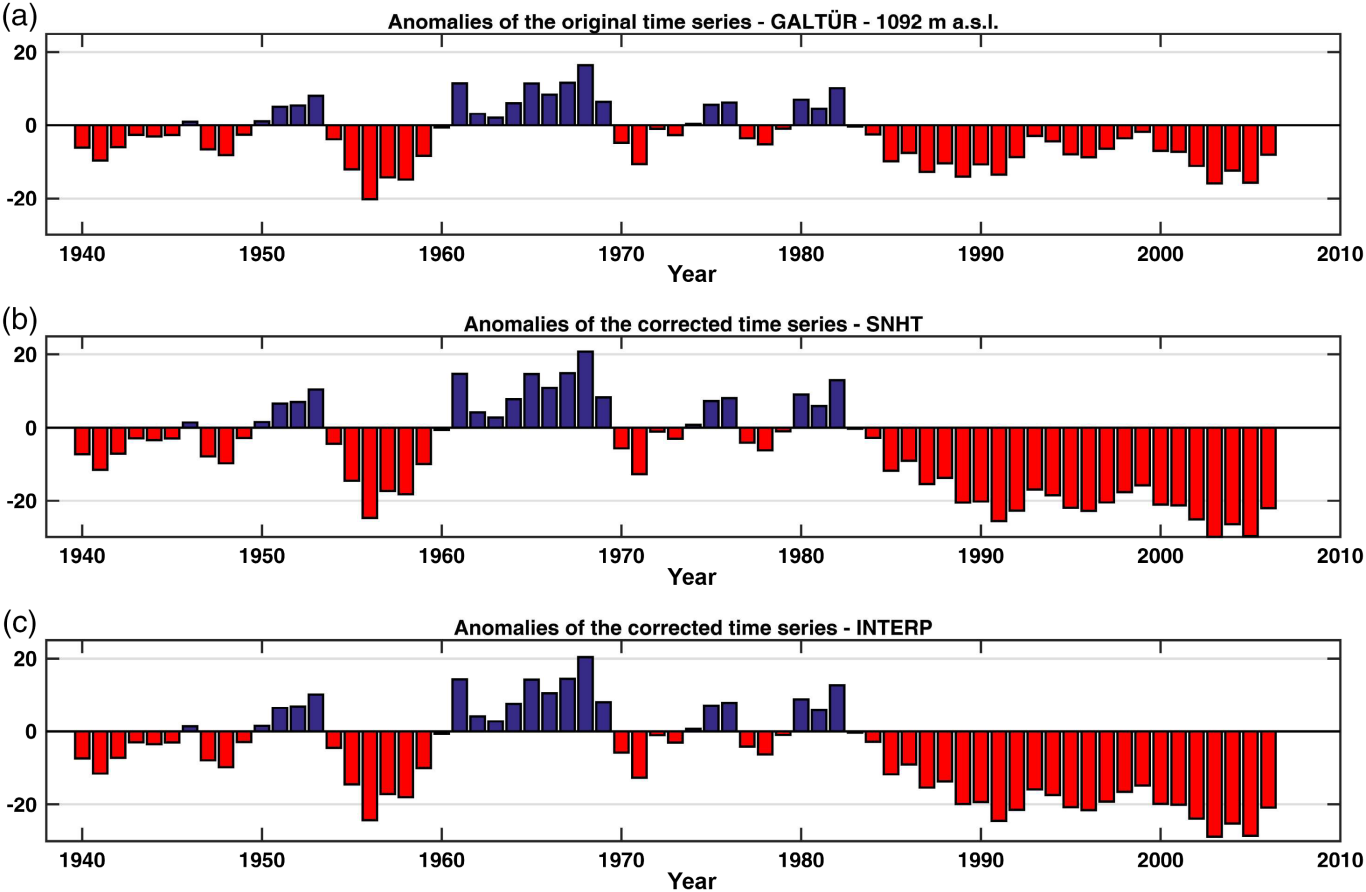
Anomalies for the period 1961–1990 of the time series of Galtür before (a) and after the adjustment of the detected breakpoint in 1988 ((b) for SNHT and (c) for INTERP). Note that the adjustment factor is applied to the part of the time series preceeding the location of the breakpoint

Figure 5 shows the results for the station of Galtür. The upper panel shows the anomalies with respect to the period 1961–1990 of the original time series, after smoothing with a 5‐year moving average. The second and third panels show the anomalies of the time series after the adjustments computed by the SNHT and INTERP methods, respectively. The anomalies of the adjusted time series are very similar because the values of the adjustment factors are very close to each other (1.22 for INTERP and 1.24 for the SNHT). Both adjusted time series show more pronounced negative anomalies starting from the late 1980s than the original (before adjustment) series. The Alpine region is comprised of regions that are characterized by different variability in the snow depth due to several factors such as location, elevation and influence of different weather patterns. However, the anomalies of the adjusted time series described above are more consistent with the results of other studies when referring to the same time period in the Alpine region (see e.g., Beniston *et al.,* 2003; Marty, 2008; Valt and Cianfarra, 2010; Marcolini *et al.,* 2017a) than the original time series.

**Figure 6:**
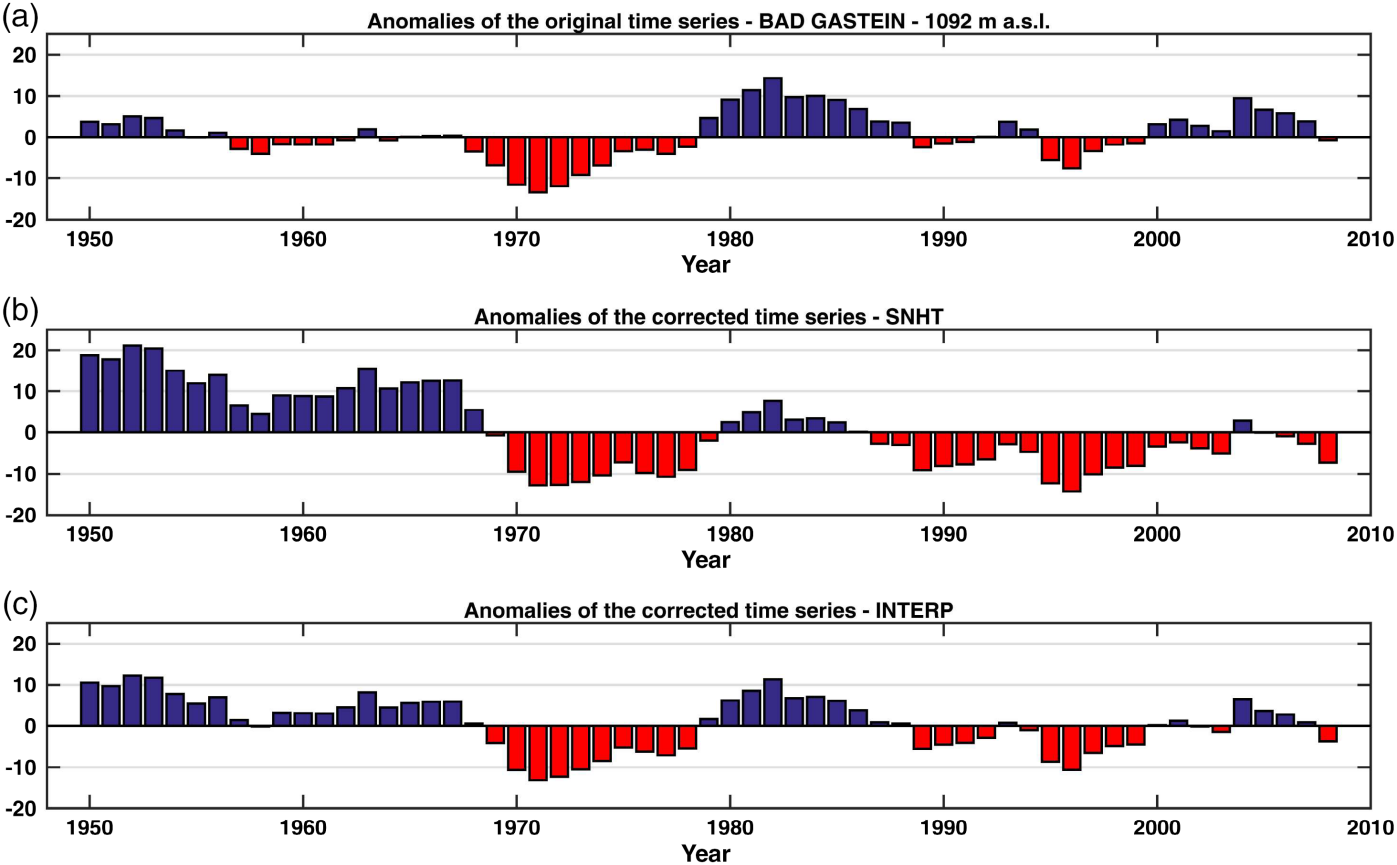
Anomalies for the period 1961–1990 of the time series of Bad Gastein before (a) and after the adjustment of the detected breakpoint in 1972 ((b) for SNHT and (c) for INTERP). Note, that the adjustment factor is applied to the part of the time series preceeding the location of the breakpoint

Another interesting example of how the adjustment of a time series can influence a climatological analysis is shown in Figure 6, where the anomalies of the Bad Gastein time series before and after the adjustment are shown. All anomalies in this case were also affected by the breakpoint adjustment and were smoothed using a 5‐year moving average. In the original time series, we see two periods with large anomalies in the 1970s (negative) and in the 1980s (positive). Furthermore, a slight increase in the snow depth after 2000 is observed. After the adjustment of the time series with the factors computed by INTERP, there are some remarkable differences. Large positive anomalies appear from the beginning of the time series until the late 1960s. The corrected time series also display negative anomalies from the late 1980s until 2000, with the following period characterized by little variability. A similar pattern is also seen in the time series adjusted by the SNHT, although the negative anomalies starting at the end of the 1980s are more evident than in the previous case and continue until present day with the exception of 2004. The positive anomalies at the beginning of the 1980s are very small in comparison. Again, the corrected time series are in better agreement with what was observed in previous studies, with a reduction in mean seasonal snow depth starting from the late 1980s. The homogenization analysis and the subsequent adjustment of the time series may therefore reduce the uncertainty in the detection of climate change.

**Figure 7:**
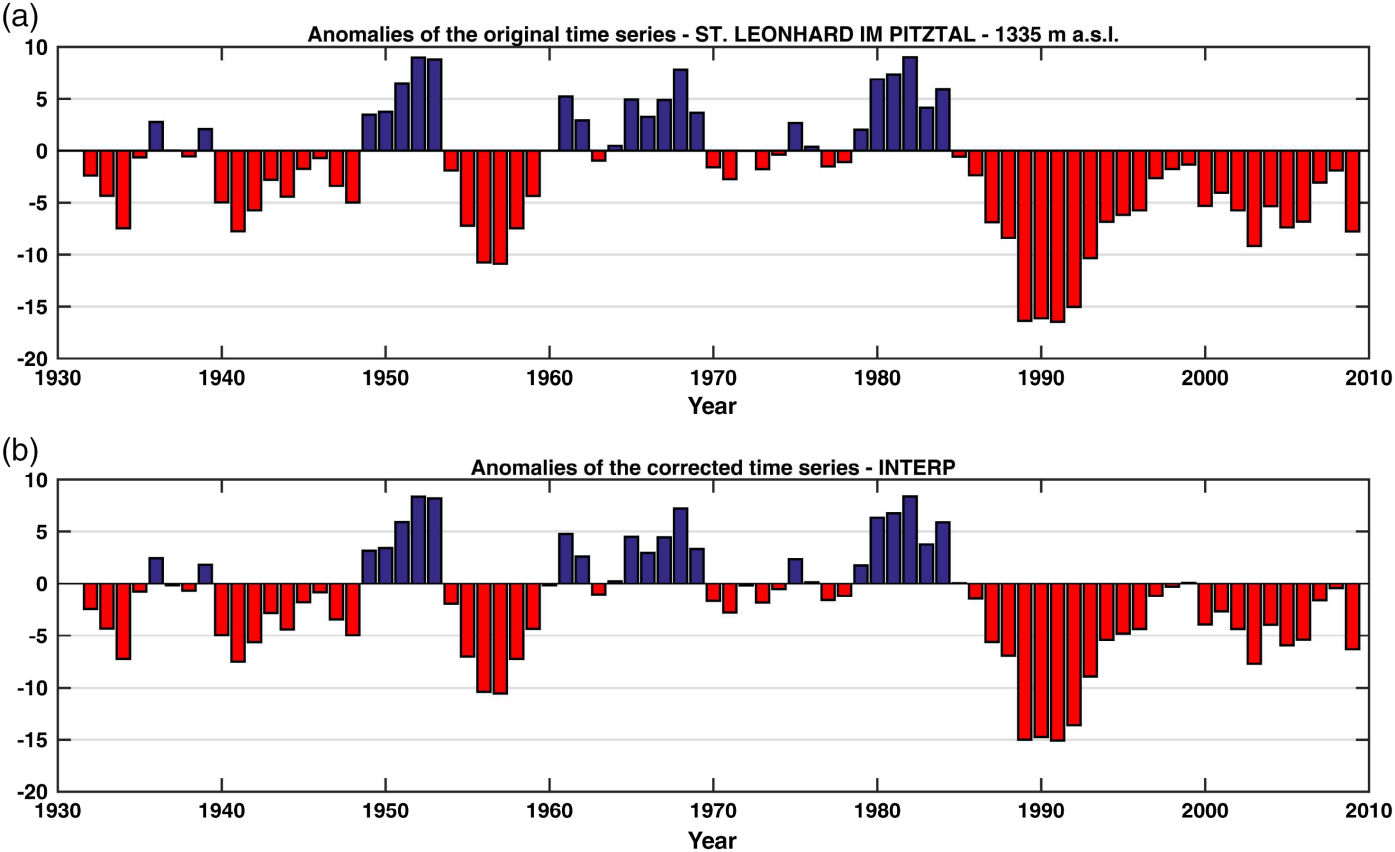
Anomalies for the period 1961–1990 of the time series of St. Leonhard im Pitztal before (a) and after the adjustment (INTERP) of the detected breakpoint in 1985 (b). Note that the adjustment factor is applied to the part of the time series preceeding the location of the breakpoint

If the value of the adjustment factors is close to 1, the correction of the inhomogeneity has little influence on climatological analysis. This is the case for the St. Leonhard im Pitztal time series shown in Figure 7.

Even after the application of the adjustment factor, the anomalies of the time series of Galtür, Bad Gastein and St. Leonhard im Pitztal (Figures 5, 6, 7) differ from each other due to the different characteristics of the area in which the stations are located (e.g., the altitude, the regional variability). This is important in order to characterize the relationship between snow depth and other related climatic and environmental factors.

### 4.5 Implication for trend analysis

The homogenization of a time series influences the results of the statistical tests that are commonly used in time series analysis. In this study, the Mann–Kendall non‐parametric statistical test (Mann, 1945; Kendall, 1975) was used to detect the presence of any decreasing or increasing trend in the snow depth time series. In addition, the Theil–Sen method (Sen, 1968) is applied to estimate the slope magnitude of the trend line (strength of the trend). Table 4 shows that 40% of the original time series display a significant negative trend at a 95% confidence level. The results obtained after the correction of the time series using HOMOP and SNHT change the percentage of time series with significant negative trends to 44% in both cases. In five cases, the adjusted time series display the same negative trend as the original series (Weitra, Galtür, St. Leonhard im Pitztal, Tamsweg, Weitensfeld), but the *p*‐value of the test and of the Sen slope generally shifts towards a more significant negative trend. The homogenized time series show a different trend (from positive to negative) in comparison to the original time series for the stations Bad Gastein, Rauris, Göstling an der Ybbs and Oed, although the *p*‐value is larger than .05. Several studies have identified the occurrence of negative trends in snow related variables throughout the entire Alps (Beniston *et al*. (2003); Marty, 2008; Valt and Cianfarra (2010); Marcolini *et al*. (2017b)), and particularly in Austria (Schöner *et al*. (2019)). The set of homogenized time series using the SNHT method leads to the identification of more time series displaying a more negative trend than the set of homogenized time series obtained using HOMOP. However, since the dataset is limited, neither of the two methods can be determined to be superior to the other. Moreover, the interpretation of the results shown in Table 4 depends on the chosen confidence level. Our analysis aims at highlighting any uncertainty that may affect the trend analysis of snow depth data, depending on whether a homogenization technique is applied or not. While the detection of breakpoints in a time series is of unquestionable importance as a quality‐check of the available dataset, the inclusion of adjusted time series for this kind of analysis is still a matter of discussion (Marty 2008; Marcolini *et al*. 2017a).

**Table 4:** Theil–Sen slope (cm/season) and results of the Mann–Kendall trend test for all 25 time series

Site	Original		HOMOP		SNHT	
	Sen slope	p‐value	Sen slope	p‐value	Sen slope	p‐value
*Galtür*	−0.410	.086	−0.866	**.002**	−0.893	**.002**
Innerkrems	−0.569	**.002**	−0.569	**.002**	−0.569	**.002**
Kals	−0.362	**.008**	−0.362	**.008**	−0.362	**.008**
*St. Leonhard im Pitztal*	−0.240	.068	−0.192	.112	−0.240	.068
Holzgau	−0.442	**.031**	−0.442	**.031**	−0.442	**.031**
*Bad Gastein*	0.082	.575	−0.033	.711	−0.233	.131
Umhausen	−0.205	**.029**	−0.205	**.029**	−0.205	**.029**
*Tamsweg*	−0.130	.074	−0.090	.123	−0.130	.074
Schönberg im Stubaital	−0.104	**.046**	−0.104	**.046**	−0.104	**.046**
Untertauern	−0.304	.160	−0.304	.160	−0.304	.160
*Rauris*	0.044	.660	−0.155	.238	−0.273	.064
Kelchsau	−0.214	.054	−0.214	.054	−0.214	.054
Ötz	−0.099	**.010**	−0.099	**.010**	−0.099	**.010**
Schladming	−0.118	.094	−0.118	.094	−0.118	.094
*Weitensfeld*	−0.323	**.000**	−0.323	**.000**	−0.168	**.004**
Mayrhofen	−0.204	**.010**	−0.204	**.010**	−0.204	**.010**
Almsee	−0.049	.738	−0.049	.738	−0.049	.738
*Weitra*	0.026	.255	0.026	.255	0.059	**.016**
Jenbach	−0.139	**.003**	−0.139	**.003**	−0.139	**.003**
*Göstling an der Ybbs*	0.069	.503	0.069	.503	−0.000	1.000
Kufstein	−0.143	.063	−0.143	.063	−0.143	.063
Frankenfels	−0.083	.195	−0.083	.195	−0.083	.195
Klagenfurt	−0.159	**.002**	−0.159	**.002**	−0.159	**.002**
*Oed*	0.001	.980	−0.010	.581	−0.039	.101
Wien Hohe Warte	−0.031	.130	−0.031	.130	−0.031	.130

*Note*: Positive (negative) Theil–Sen slopes indicate increasing (decreasing) tendency. Bold values indicate a statistical significance at a 95% confidence level. The corrected station time series are highlighted in italics (left column). The trend test is valid for the winter seasons from NDJFM between 1961 and 2010.

## 5 CONCLUSIONS

To the best of our knowledge, this is the first study to compare two snow depth homogenization algorithms.

The main outcome of this work shows that homogenization of snow depth time series is possible and necessary for climatological analysis. This conclusion is drawn after applying two different homogenization methods (SNHT and HOMOP) to time series from a number of Austrian stations.

The dataset consists of manual snow depth measurements; hence, it was not possible to identify the influence of the measurement method on the homogeneity of the time series. We believe that this constitutes an important advantage for the analysis we performed, since it allows a focus on the comparison of the two methods for a relatively simple situation, where breakpoints are mainly caused by station relocation. As shown in Marcolini *et al*. (2017a), merging datasets collected using different methods can also be an important source of inhomogeneity in the time series and should be investigated further in future studies.

The detection of breakpoints in mean seasonal snow depth time series is possible since HOMOP and SNHT agree in most cases, showing a good reliability in localizing breakpoints. The biggest challenge encountered was that HOMOP sometimes detected suspicious breaks during particularly snow‐scarce seasons in low elevation sites, while SNHT classified the time series as homogeneous. Our recommendation to solve this issue is to verify the occurrence of the break using either metadata or a different homogenization method. The identification of possibly suspicious breaks is a matter of great importance to prevent unneeded adjustments of the time series, which already represent the “true” variation of the snow depth.

For both algorithms, it is vital to compare the statistical results with the metadata, that is, all the available documentation of changes that are not due to natural factors and that may have affected the measurements such as relocation of the station or change in the measuring equipment. We suggest that if the detected breakpoints cannot be confirmed with the metadata, the classification of the time series as inhomogeneous should be supported by further robust arguments such as the detection of the same breakpoint using different homogenization methods.

The adjustment factors computed by INTERP and SNHT are generally in agreement, although the available sample is too small to derive statistically significant conclusions. As it has also been discussed in Peterson *et al*. (1998), the adjustment of an inhomogeneous time series is not straight forward and it is more difficult than the identification of a breakpoint. This study further highlights how the magnitude of the homogenization factors is method‐dependent. It is important to remember that homogenized time series are not equivalent to homogeneous time series (Peterson *et al.,* 1998) as their adjusted values are not uniquely defined. The consideration or the exclusion of adjusted time series for climatological analysis is therefore subjective. The homogenized part of the time series must be considered a statistically justified estimate of the true values.

The homogenization of mean seasonal snow depth time series is necessary as it affects both the evaluation of the anomalies, trend analysis performed with Mann–Kendall tests and the estimation of the Theil–Sen slope. The homogenized dataset showed an increase in the number of stations with negative snow depth trends and with consecutive negative anomalies starting from the late 1980s and early 1990s, which was in agreement with the observations available for several stations in the Alps.

In this work, we purposefully did not rank the performance of the two methods. As also outlined in Marcolini *et al*. (2017a), it is not always appropriate to rank the different homogenization algorithms in terms of performance as they are statistical tests and they are inherently affected by uncertainty. As a general suggestion, we should not rely on the results of a single test in the case of mean seasonal snow depth time series due to the inherent complexity in the homogenization of these datasets. Instead, we recommend the application of multiple and fundamentally different tests to verify the reliability of the identified breakpoints and their correction factors. A breakpoint identified by different methods is more reliable than a breakpoint that emerges from only a single test, that is, only under the specific statistical assumptions on which the test is based.

It is quite clear, though, that further studies are needed to improve the capability of the breakpoint detection algorithm to identify artificial shifts in snow depth time series. With the methods used in this study, daily snow depth data cannot be corrected, as the multiplicative correction factor does not generate new snow days. Hence, further research is needed to develop robust methods to correct daily snow depth time series. In the future, it would also be interesting and important to test the performances of other homogeneity tests on a snow depth dataset and compare their outcomes with the results of PRODIGE and the SNHT.
